# A Rare Case of Ventilator-Associated Pneumonia Caused by Cupriavidus Pauculus

**DOI:** 10.7759/cureus.8573

**Published:** 2020-06-12

**Authors:** Syed A Huda, Sanjay Yadava, Sara Kahlown, Mohammad H Jilani, Bashar Sharma

**Affiliations:** 1 Internal Medicine, State University of New York (SUNY) Upstate Medical University, Syracuse, USA; 2 Internal Medicine, United Hospital, Johnson City, USA; 3 Internal Medicine, VA Medical Center (VAMC), Syracuse, USA

**Keywords:** vap, cupriavidus pauculus, icu, intubated

## Abstract

Ventilator-associated pneumonia is a hospital-acquired infection that is commonly encountered in intubated patients in the intensive care unit (ICU). It is associated with significant morbidity and mortality. The causative organisms include gram-negative rods (Escherichia coli, Klebsiella pneumoniae, or Acinetobacter species) or gram-positive cocci (Staphylococcus aureus). Described here is a case of ventilator-associated pneumonia caused by a relatively unknown gram-negative bacterium, Cupriavidus (C.) pauculus that was successfully treated with intravenous cefepime.

## Introduction

Ventilation-associated pneumonia (VAP) is a very common, life-threatening hospital-acquired infection in patients who have been intubated in the intensive care unit (ICU) [1]. It is difficult to determine the accurate incidence of VAP due to the absence of a gold standard test and great variation in the methods employed for diagnosis. As per the latest report by the US National Healthcare Safety Network (NHSN), the mean VAP rate in North America was one to 2.5 cases per 1000 ventilator days. It is much lower than the incidence rate reported in Europe [2]. The most common pathogens causing VAP include gram-negative bacteria Escherichia coli, Pseudomonas aeruginosa, Klebsiella pneumoniae, or Acinetobacter species while Staphylococcus aureus is the predominant gram-positive bacteria [3]. Here, we report VAP caused by a lesser-known gram-negative bacterium called Cupriavidus (C.) pauculus.

## Case presentation

A 41-year-old female with a medical history significant for intravenous drug abuse (IVDU) presented to the emergency department (ED) with two months history of flu-like symptoms, malaise, weakness, weight loss, shortness of breath on exertion and fever for one week. On presentation, she was in septic shock not responding to fluid resuscitation, so she was started on pressor support and also intubated for hypoxemia. She was also hypoxic, so she had to be intubated. She was treated empirically with IV vancomycin and piperacillin-tazobactam while sepsis workup, including blood, sputum, and urine cultures, were pending. Her chest X-ray (CXR) showed multiple septic emboli in bilateral lungs and echocardiogram revealed large mobile vegetation attached to the anterior tricuspid valve. Her urine and blood cultures drawn on admission grew methicillin-sensitive Staphylococcus aureus (MSSA). She was diagnosed with infective endocarditis and her antibiotics were switched to IV cefazolin based on culture sensitivities. Her condition improved initially with repeat blood cultures on Day 3 showing no growth. However, she developed fever along with increased oxygen demand and sputum production on Day 5 of intubation so blood, urine, and sputum cultures, as well as CXR, were repeated. Imaging was inconclusive for new consolidation due to extensive bilateral septic emboli. However, gram staining of tracheal aspirate showed 2+ white blood cells and gram-negative bacilli, which were later identified as non-lactose fermenting C. pauculus. Although the imaging study was indeterminate, other clinical findings of new onset of fever, worsening hypoxia, leukocytosis, and positive tracheal aspirate culture were consistent with ventilator-associated pneumonia. Her antibiotic was switched to IV cefepime and it was continued based on sensitivity results. Her fever and leukocytosis resolved gradually and ventilatory requirements improved; hence, cefepime was switched back to IV cefazolin for MSSA coverage after a course of seven days. The patient was extubated on Day 12 of hospital admission with the gradual improvement of her respiratory status and finished four weeks of IV antibiotics and underwent successful tricuspid valve replacement.

## Discussion

Ventilator-associated pneumonia (VAP) is a type of hospital-acquired pneumonia that develops after more than 48 hours of mechanical ventilation. VAP is a common and serious problem in the intensive care unit and is associated with an increased risk of death. VAP can be caused by a wide variety of bacteria that originate from the patient’s flora or the healthcare environment. It can be monomicrobial or polymicrobial. Common pathogens include aerobic gram-negative bacilli (eg, Escherichia coli, Klebsiella pneumoniae, Enterobacter spp, Pseudomonas aeruginosa, Acinetobacter spp) and gram-positive cocci (eg, Staphylococcus aureus, including methicillin-resistant S. aureus (MRSA), Streptococcus spp) with a low prevalence of Serratia spp, Stenotrophomonas maltophilia, and community-acquired pathogens [4-5]. C. pauculus is a rare cause of VAP, with only two cases reported in English literature [6-7]. C. pauculus is a non-lactose, fermenting, gram-negative, non-spore-forming rod with positive oxidase and catalase reactions. C. pauculus was initially classified in the genus Ralstonia. It was re-classified in the genus Wautersia in 2004. In the same year, the genus was renamed as Cupriavidus [8]. It is commonly found in soil, water, or plants. It has been implicated in pseudo-outbreaks of skin and superficial site infections. It is believed to cause nosocomial infections by the contamination of nebulization solution, ultrafiltrate water, thermos-regulator reservoir water, extracorporeal membrane oxygenation system (ECMO), tap water, hydrotherapy pools, and bottled mineral water [9-11]. It causes infection in predominantly immunocompromised hosts, especially in hematological malignancies and hematopoietic stem cell transplant (HSCT) patients. However, there are case reports of *C.* pauculus causing infection in healthy hosts. Limited numbers of intra-abdominal infection, meningitis, tenosynovitis, and community-acquired pneumonia cases have been reported in different age groups with different immune status [12]. Our patient was immunocompetent but critical illness due to MSSA bacteremia/endocarditis with metastatic foci of infection in lungs, most likely placed her at a higher risk of infection. It is difficult to comment on the choice of antibiotics for the infection although the pathogen has been successfully treated with the third and fourth generation of cephalosporins especially cefepime, ceftazidime, cefotaxime, and carbapenem as monotherapy or in combination with aminoglycosides or ciprofloxacin.

## Conclusions

In conclusion, C. pauculus is an environmental bacteria that rarely causes infection in immunocompetent humans. Isolation from a clinical specimen should be treated with a high index of suspicion in the appropriate clinical settings. To our knowledge, this is the third case of VAP caused by C. pauculus.
